# Flaxseed-enriched diets change milk concentration of the antimicrobial danofloxacin in sheep

**DOI:** 10.1186/s12917-018-1341-3

**Published:** 2018-01-15

**Authors:** Jon Andoni Otero, Dafne García-Mateos, Indira Alvarez-Fernández, Rocío García-Villalba, Juan Carlos Espín, Ana Isabel Álvarez, Gracia Merino

**Affiliations:** 1 Department of Biomedical Sciences-Physiology, Veterinary Faculty, University of Leon, 24071 Leon, Spain; 2 Instituto de Desarrollo Ganadero y Sanidad Animal (INDEGSAL), University of Leon, 24071 Leon, Spain; 3 Laboratory of Food and Health, Research Group on Quality, Safety and Bioactivity of Plant Foods, Department of Food Science and Technology, CEBAS-CSIC, 30100 Murcia, Spain

**Keywords:** Danofloxacin, Mammary, Flaxseed, Enterolactone, Enterodiol, Milk residues

## Abstract

**Background:**

Flaxseed is the most common and rich dietary source of lignans and is an acceptable supply of energy for livestock. Flaxseed lignans are precursors of enterolignans, mainly enterolactone and enterodiol, produced by the rumen and intestinal microbiota of mammals and have many important biological properties as phytoestrogens. Potential food-drug interactions involving flaxseed may be relevant for veterinary therapy, and for the quality and safety of milk and dairy products. Our aim was to investigate a potential food-drug interaction involving flaxseed, to explore whether the inclusion of flaxseed in sheep diet affects concentration of the antimicrobial danofloxacin in milk.

**Results:**

Increased concentrations of enterodiol and enterolactone were observed in sheep plasma and milk after 2 weeks of flaxseed supplementation (*P* < 0.05). However, enterolactone and enterodiol conjugates were not detected in milk. Milk danofloxacin pharmacokinetics showed that area under the curve (AUC)_0–24,_ maximum concentration (C_max_) and AUC_0–24_ milk-to-plasma ratios were reduced by 25–30% in sheep fed flaxseed-enriched diets (*P* < 0.05). Our results demonstrate, therefore, that flaxseed-enriched diets reduce the amount of danofloxacin in sheep milk and enrich the milk content of lignan-derivatives.

**Conclusion:**

These findings highlight an effect of flaxseed-enriched diets on the concentration of antimicrobials in ruminant’s milk, revealing the potential of these modified diets for the control of residues of antimicrobial drugs in milk.

## Background

**Fig. 1:**
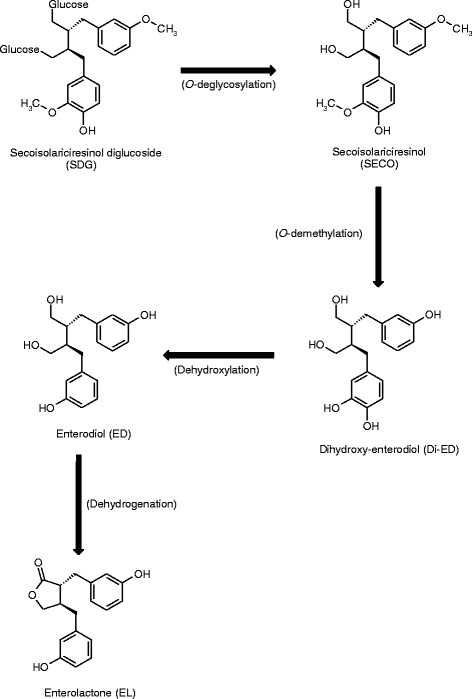
Main metabolic pathways from plant lignans (secoisolariciresinol diglucoside and secoisolariciresinol) to enterolignans (dihydroxy-enterodiol, enterodiol and enterolactone) catalyzed by gut microbiota. Adapted from Mukker et al. [3]

Flaxseed is the main source of lignans [1] and whole flaxseed is an attractive ingredient for cow rations as a source of energy and protein [2]. Secoisolariciresinol diglucoside (SDG), the main flax lignan, is metabolized by ruminal and intestinal microbiota, which leads to the formation of mammalian enterolignans, enterodiol and enterolactone [3] (Fig. 1). Finally, conjugation of enterolignans, mainly with sulfate and glucuronic acid, occurs in the digestive tract and liver [4, 5].

Flax lignans are important for the veterinary industry since they improve survival of cow embryos [6], they improve ruminal metabolism in goats [7] and they can protect against oxidative stress damage in the mammary gland [8]. Moreover, lignans and their derivatives provide many human health benefits against menopausal symptoms, breast and prostate cancers, cardiovascular diseases, osteoporosis and diabetes [9], with biological activities at nutritionally and physiologically relevant concentrations, even in the nM range [10, 11]. Flaxseed has become an attractive ingredient in human diets specially designed for specific health benefits [12] and has been incorporated in several dietary items including milk and dairy products [13].

However, some authors have advised caution when flaxseed is consumed during pregnancy and lactation due to changes in milk and body composition [14] and in hormonal and biochemical parameters [15]. In addition, societies are increasingly demanding assurance of safety and efficacy in drugs used on animals, and in foodstuffs derived from food-producing species [16], all of which restricts the diversity of drugs that are approved for the treatment of infectious diseases [17]. In this context, potential food-drug interactions have to be carefully considered. Previous studies have in fact addressed the effects of dietary compounds, such as soy isoflavones, on the amount of antimicrobial residues in milk [18, 19]. This is a very relevant issue regarding to food safety, where the potential milk contamination by fluoroquinolones is particularly significant, since these antimicrobials are drugs with considerable stability when subjected to thermal and cooking procedures. Consequently, fluoroquinolones can remain in milk after dairy processing, and thus can reach consumers [20]. Likewise, intermittent exposure to low levels of fluoroquinolones can produce hypersensitivity reactions or affect the intestinal microbiota [21–23] or contribute to the development of antibiotic resistance in dairy consumers. A strict framework exists to control the use of antimicrobials. In the case of the fluoroquinolone danofloxacin, which is used for the treatment of mastitis and respiratory infections in ruminants, the maximum residue limit in milk is 30 μg/kg [24].

The objective of the present study was to determine whether supplementation of dairy sheep with whole flaxseed affects the milk concentrations of the fluoroquinolone danofloxacin. In addition, plasma and milk levels of enterolignans (enterodiol and enterolactone) and their glucuronide and sulfate conjugates were analyzed for the first time in sheep fed flaxseed-enriched diets.

## Methods

This experiment was conducted at the Experimental Farm of the University of Leon (Spain). Animals were housed and handled according to institutional guidelines complying with European legislation [25].

### Experimental design and pharmacokinetic studies

Eighteen lactating Assaf sheep (3–4 months in lactation and weighing 66–105 kg) were used and milked twice daily. The animals were parasite-free, and drinking water was available ad libitum. The experimental design was performed with animals stratified according to milk production and number of days post-partum, and then randomly distributed in the experimental groups. The animals were divided into 3 diet groups of 6 animals each. One group of sheep was fed a standard diet, a second group was fed a lignan-enriched diet containing 10% whole flaxseed (Nutricor SL, Leon, Spain) for 2 weeks, and a third group was fed a lignan-enriched diet containing 15% whole flaxseed (Nutricor SL) also for 2 weeks (diet composition is shown in Table 1). Flaxseed amounts and duration of treatment were chosen based on previous studies on cows [2, 26]. Whole flaxseed composition included 20.2% of crude protein, 13.1% of crude fiber and 28.5% of ether extract. All sheep were fed ad libitum twice per day after each milking (09.00 and 18.00 h). Feed intake was not affected by the different experimental diets.

**Table 1:** Composition of the diets (as fed-basis, otherwise indicated)

Ingredients/analysis, %	Control	Flaxseed 10%	Flaxseed 15%
dehydrated alfalfa hay	40.0	40.0	40.0
whole flaxseed	0.0	10.0	15.0
corn grain	18.6	18.6	15.0
rape seed	13.7	11.0	10.0
barley grain	6.3	5.0	5.6
beet pulp	6.0	3.0	3.0
oat grain	5.0	3.0	5.0
cotton seed	5.0	4.0	1.0
Molasses	4.0	4.0	4.0
vitamin and mineral supplements	1.4	1.4	1.4
dry matter	90.90	91.02	91.08
neutral detergent fibre, % DM	30.79	29.33	29.04
crude protein, % DM	18.06	18.05	18.05
Energy (Mj/kg)	11.89	12.14	12.21

After 2 weeks on the experimental diets, animals in all groups received a single therapeutic dose (1.25 mg/kg, i.m.) of danofloxacin (Advocin 2.5%, Pfizer, Cedex, France). Blood samples (5 mL) were collected from the jugular vein in commercial blood collection tubes (Vacutainer, 10 mL; Becton Dickinson, Franklin Lakes, NJ) before treatment, and at 0.5, 1, 2, 4, 6, 8, 10, 12, and 24 h after danofloxacin administration. The pharmacokinetic schedule was based on previous studies from our group [19, 27, 28]. Plasma was separated by centrifugation at 1000 x *g* for 15 min and stored at − 20 °C until analysis. Milk samples were collected using an automated milking machine, before treatment, and at 0.5, 1, 2, 4, 6, 8, 10, 12, and 24 h after danofloxacin administration. A complete evacuation of the udder was carried out at each sampling to avoid any dilution effect. Samples were stored at − 20 °C until analysis.

### Analysis of Enterolignans and related compounds

To obtain an estimation of the overall lignan dose and the amount of lignan-derivatives in milk, plasma and milk samples collected after 2 weeks of flaxseed supplementation were analyzed to determine the concentrations of the main microbiota-derived enterolignans. Plasma and milk aliquots (100 μL) were mixed with sodium acetate buffer and treated with hydrochloric acid in methanol, following the method of Bolca et al. [29]. The dry residue was dissolved in methanol and filtered (0.22 μm) prior to injection into the ultra-performance liquid chromatography quadrupole time-of-flight mass spectrometry (UPLC-QTOF-MS) equipment.

Analyses were developed using an Agilent 1290 Infinity LC system coupled to a 6550 Accurate-Mass QTOF (Agilent Technologies, Waldbronn, Germany) and using an electrospray interface (Jet Stream Technology). Analytical conditions were as previously described with some modifications [30]. Briefly, 5 μL of sample was injected onto a reverse phase Poroshell 120 EC-C18 column (3 × 100 mm, 2.7 μm) (Agilent Technologies) operating at 30 °C. Water with 0.1% formic acid (A) and acetonitrile with 0.1% formic acid (B) were used as mobile phases at a flow rate of 0.4 mL/min and the following gradient: 5–25% B at 0–10 min, 25–40% B at 10–20 min; 40–90% B at 20–24 min; 90%–5% B at 25–26 min and the column re-equilibrated for 4 min. The electrospray interface parameters were as follows: gas temperature, 280 °C; drying gas flow, 9 L/min; nebulizer pressure, 35 psi; sheath gas temperature, 400 °C; sheath gas flow, 12 L/min. Spectra were acquired in the *m/z* range from 100 to 1100 in negative mode and fragmentor voltage was 100 V.

Data were processed using Mass Hunter Qualitative Analysis software (version B.06.00 Agilent Technologies). A target screening strategy was applied to plasma and milk samples for the qualitative screening of possible enterolignan-related metabolites. A list of target compounds (60 possible metabolites) covering different phase I and phase II metabolites of parent compound and microbial metabolites was sought in all the samples after MS full-acquisition. Screening was based on mass filtering at the exact mass of the studied compound using narrow mass extraction windows (0.01 *m/z*). Identification was possible from the valuable information given in QTOF-MS acquisition mode which provides possible molecular formulae for the compounds based on accurate mass and isotopic pattern (Table 2). Additionally, a targeted MS/MS experiment offered fragmentation information for greater reliability in the compound identification process. When possible, direct comparison with authentic standards was performed. Quantification of enterolactone and enterodiol was determined by interpolation in the calibration curve obtained from their own available standards. These metabolites were quantified in MS by peak area integration of their extracted ion chromatograms. Calibration curves were linear from the limit of quantification (LOQ) to 500 nmol/L. LOQ values were 0.36 nmol/L for enterolactone and 0.65 nmol/L for enterodiol. In the case of conjugated metabolites accurate quantification was not possible due to the absence of available standards and so only qualitative results are provided.

**Table 2:** Enterolignans and related conjugates identified by UPLC-QTOF-MS analysis in plasma and milk of sheep fed with flaxseed-enriched diets

Compound	Localization	Retention time (min)	*m/z*	Molecular formula	Error	Score	MS/MS fragments
Enterodiol	Plasma, milk	13.614	301.1443	C_18_H_22_O_4_	0.89	99.51	271/253/241/146
Enterodiol- glucuronide	Plasma	10.422	477.1761	C_24_H_30_O_10_	0.67	89.58	301/253/175/113
Enterodiol- sulfate	Plasma	11.055	381.1010	C_18_H_22_O_7_S	0.53	92.51	301/253/107
Enterolactone	Plasma, milk	17.550	297.1132	C_18_H_18_O_4_	−0.07	98.81	253/189/165/121/107
Enterolactone-glucuronide	Plasma	12.437/12.618	473.1457	C_24_H_26_O_10_	−0.44	98.03	297/253/175/113
Enterolactone-sulfate	Plasma	13.315	377.0701	C_18_H_18_O_7_S	0.07	95.95	297/253/107

### HPLC analysis of Danofloxacin

Conditions for HPLC analysis of danofloxacin concentrations in plasma and milk were modified according to previously published methods [27, 31]. Difloxacin (Sigma-Aldrich) at 0.1 μg/mL for the plasma sample extraction and 0.01 μg/mL for the milk sample extraction was used as internal standard. To each 100 μL aliquot of sample, 600 μL of chloroform was added. Samples were shaken and the organic and water phases were separated by centrifugation at 5000 x *g* for 6 min and evaporated to dryness under a nitrogen stream. The residue was reconstituted in 100 μL of methanol and injected into the HPLC system. The system consisted of a Waters 600 pump, a Waters 717 plus autosampler, and a Waters 486 fluorescence detector (Waters Corporation, Milford, MA). Separation of the analytes was achieved with a C_18_ reversed-phase column (Mediterranea Sea 18 5 μm 250 × 0.46 mm; Teknokroma, Barcelona, Spain). The mobile phase consisted of 25 m*M* orthophosphoric acid supplemented with 0.1% triethylamine (pH 3.0): acetonitrile (80:20 *v*/v) at a flow rate of 1.6 mL/min. Sample detection was performed by fluorescence detection at 338 nm (excitation) and 425 nm (emission). Integration was carried out using MILLENNIUM 32 software (Waters). Standard samples in the appropriate drug-free matrix were prepared, yielding a concentration range from 0.0019 to 0.5 μg/mL for plasma samples and 0.019 to 5 μg/mL for milk samples. Correlation coefficients (r) for danofloxacin ranged between 0.998 and 0.984 for the calibration curves in plasma and milk curves respectively. LOQ were 1.9 ng/mL for sheep plasma and 19 ng/mL for sheep milk.

### Pharmacokinetic calculations and statistical analyses

Peak concentration (C_max_) and time–peak concentrations (T_max_) were read from the plotted concentration–time curve for each animal. The area under the plasma concentration–time curves (AUC) was calculated using the linear trapezoidal rule. Mean residence time (MRT) was calculated by the linear trapezoidal rule with extrapolation to time infinity, using the formula: MRT = AUMC/AUC, where AUMC is the mean area under the first moment curve. These calculations were made with a computer program (PK solution 2.0, Summit Research Services, Ashland, OH) and determined by non-compartmental analyses.

Results are presented as the mean ± standard deviation (SD). Normal distribution of data was analyzed by the Shapiro-Wilk test. Comparisons between groups were carried out by analysis of variance (ANOVA) followed by the Newman–Keuls test for normally distributed data, while Kruskal-Wallis test followed by the Dunn-Bonferroni correction was used when a non-parametric test was required. A probability of *P* < 0.05 was considered to be statistically significant.

## Results

### Plasma and milk levels of enterolignans

**Fig. 2:**
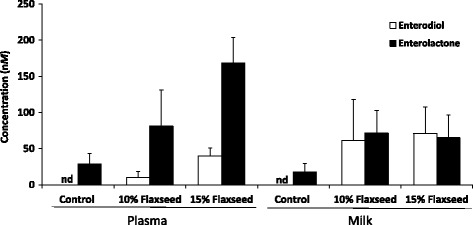
Enterodiol and enterolactone concentrations (nM) in plasma and milk in animals fed flaxseed-enriched diets for two weeks in control diet group (*n* = 6), 10% flaxseed-enriched diet group (*n* = 6) and 15% flaxseed-enriched diet group (*n* = 6). Samples were taken just before danofloxacin administration coinciding with the morning milking. The results are presented as means; error bars indicate SD

Enterodiol and enterolactone were identified in plasma and milk (Table 2). Enterodiol was present in plasma and milk in sheep fed flaxseed, whereas basal levels of enterolactone were also present in the groups fed standard diet (Fig. 2). The concentrations of both enterolactone and enterodiol increased after 2 weeks of flaxseed supplementation (*P* < 0.05, except for enterolactone in plasma from the group fed 10% flaxseed).

As conjugation of enterolignans, mainly with sulfate and glucuronic acid, occurs in the digestive tract and liver [4, 5], enterolignan metabolites were also analyzed. In addition to enterodiol and enterolactone, their glucuronide and sulfate conjugates (enterolactone-glucuronide, enterodiol-glucuronide, enterolactone-sulfate and enterodiol-sulfate) were identified after the targeted analysis in plasma of the group with 15% flaxseed (Table 2). However, due to the absence of available standards, accurate quantification of the levels of these compounds was not possible. Enterolactone and enterodiol conjugates were not detected in milk.

### Plasma pharmacokinetics and milk levels of danofloxacin

**Fig. 3:**
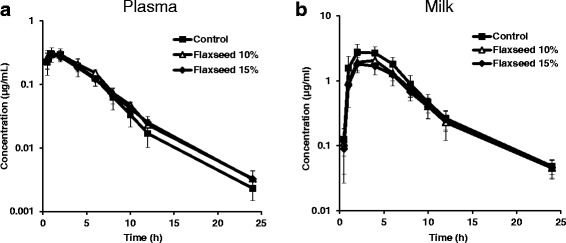
Semilogarithmic plot of plasma (**a**) and milk (**b**) concentrations of danofloxacin after its i.m. administration at a dosage of 1.25 mg/kg to sheep fed control diet (*n* = 6) (■), 10% flaxseed-enriched diet (*n* = 6) (△) and 15% flaxseed-enriched diet (*n* = 6) (♦). Plasma samples were collected at several points over 24 h. The results are presented as means; error bars indicate SD

Our results revealed that the different diets had no effect on plasma concentration and pharmacokinetic parameters of danofloxacin (Fig. 3a, Table 3). However, milk concentrations of danofloxacin and its pharmacokinetic parameters significantly differed between groups (Fig. 3b and Table 4). Flaxseed-enriched diets significantly reduced the amount of danofloxacin in milk (Fig. 3b). AUC_0–24_ values were reduced by approximately 25–30% (Table 4). C_max_ values diminished by almost 40% in the group fed 15% flaxseed (2.95 ± 0.74 μg/mL control group vs. 1.84 ± 0.44 μg/mL 15% flaxseed group, *P* < 0.05). AUC_0–24_ milk/plasma ratios were also significantly reduced in the groups fed flaxseed-enriched diets (11.04 ± 1.95 in the control group vs. 7.81 ± 2.08 and 7.57 ± 2.10 in the 10% and 15% flaxseed groups respectively; *P* < 0.05). The MRT parameter did not reach significant differences. The groups fed 10% and 15% flaxseed showed similar results and no statistical differences were obtained for any of the parameters analyzed (*P* > 0.05).

**Table 3:** Plasma pharmacokinetic parameters (means ± SD) of danofloxacin after i.m. administration at a dosage of 1.25 mg/kg in the three experimental groups; *n* = 6

Parameter	Control	Flaxseed 10%	Flaxseed 15%
AUC _0–24,_ μg·h/mL	1.74 ± 0.37	1.92 ± 0.26	1.79 ± 0.15
AUC _0-∞,_ μg·h/mL	1.75 ± 0.38	1.93 ± 0.25	1.81 ± 0.15
C_max_, μg/mL	0.31 ± 0.05	0.31 ± 0.06	0.30 ± 0.04
MRT, h	4.22 ± 0.73	4.77 ± 0.49	4.95 ± 0.39
T_max_, h	1.50 ± 0.50	1.50 ± 0.50	1.16 ± 0.37

**Table 4:** Milk pharmacokinetic parameters (means ± SD) of danofloxacin after i.m. administration at a dosage of 1.25 mg/kg in the three experimental groups; *n* = 6

Parameter	Control	Flaxseed 10%	Flaxseed 15%
AUC _0–24_ (μg·h/mL)	19.17 ± 5.03	14.58 ± 1.79^a^	13.43 ± 3.07^a^
AUC _0-∞_ (μg·h/mL)	19.44 ± 5.12	14.86 ± 1.86^a^	13.68 ± 3.13^a^
C_max_ (μg/mL)	2.95 ± 0.74	2.20 ± 0.35^a^	1.84 ± 0.44^a^
MRT (h)	5.35 ± 0.87	5.25 ± 0.35	5.54 ± 0.29
T_max_ (h)	2.66 ± 0.94	3.00 ± 1.00	2.66 ± 0.94
AUC _0–24_ milk/plasma	11.04 ± 1.95	7.81 ± 2.08^a^	7.57 ± 2.10^a^

^a^Significantly different from the control group (*P* < 0.05)

## Discussion

The increase in the amounts of flaxseed in food has been directly related with a higher concentration of enterolactone in plasma, urine and milk in dairy cattle [4, 32]. In this study, we present for the first time a direct correlation between the amount of whole flaxseed added to the diet and the concentration of enterodiol and enterolactone found in sheep plasma. Our data showed an increase of enterodiol and enterolactone concentrations in sheep plasma and milk after 2 weeks of flaxseed supplementation. Levels of enterolactone in sheep milk were in the same range as the levels previously obtained in Holstein cows with similar diets [2]. Although enterodiol was not detected in the milk of cows [2], our data reveal that enterodiol was present in plasma and milk in sheep fed flaxseed. Differences in the analytical methods used or even in species-dependent factors such as different microbiota or metabolism could explain this discrepancy.

The similar milk concentrations of enterolignans reached with the two flaxseed-enriched diets (10 and 15%), notwithstanding the different plasma concentrations, may indicate a saturation process. It has been suggested that phytoestrogen concentration in milk is modulated through diet intake although transport capacity into milk appears to be limited by saturation [5]. In fact, the transfer of enterolignans into milk has recently been reported to be mediated by the transporter protein ABCG2 [30, 33]. ABCG2 is an active transporter apically expressed in the plasma membrane of cells, involved in the secretion into milk of many compounds [34]. It is likely that the high concentrations of enterolactone obtained in plasma from the animals fed flaxseed-enriched diet produced a saturation of the ABCG2-mediated transport of enterolactone and its related compounds into the milk.

Interactions between prescribed drugs and dietary-derived compounds should be carefully considered. Previously, the effect of soy-enriched diets containing isoflavones was reported to limit concentration of drugs, including danofloxacin, in sheep milk [18, 19]. In our case, sheep fed standard or flaxseed-enriched diets were administered danofloxacin at 1.25 mg/kg to explore the potential effect of these diets on pharmacokinetics and secretion into milk of this antimicrobial. Our analysis of plasma concentrations were in accordance with previous reports in sheep [27, 35] and reveal no effect on plasma pharmacokinetic parameters with the diets containing different amounts of flaxseed. Therefore, the systemic efficacy of this antimicrobial is not compromised with these modified diets. However, in sheep fed flaxseed diet, secretion of danofloxacin into milk was reduced by 25–30%.

One mechanistic possibility to explain this food-drug interaction observed between flaxseed and danofloxacin with reduction of the milk levels of this antimicrobial could be the involvement of the transporter protein ABCG2. In fact, one of the main enterolignans, enterolactone, has recently been reported as a good inhibitor of ABCG2 [30]. An important role of ABCG2 has been reported in the secretion of antimicrobials, including danofloxacin, into ruminants´ milk, explaining the ten times higher concentrations of danofloxacin in milk than in plasma [19, 28, 34, 36]. Characterization of the drug interactions involving this transporter contributes to avoid, control, and predict drug residues in milk [37]. Previous studies have shown that ABCG2-mediated drug/food-drug interactions can inhibit drug transport into milk without any effect in plasma concentrations [18, 19, 28]. In our case, lignan-derivatives may affect transfer of danofloxacin into milk, inhibiting ABCG2. Other components of flaxseed, such as flavonoids, could also inhibit ABCG2 [38] although their concentrations in flaxseed are lower than lignan precursors [39]. In any case, a significant overlap with other transporters and metabolic pathways and competing processes of distribution and elimination clearly indicates the challenge of identifying a drug/food-drug interaction as a result of inhibition of ABCG2 [40].

Factors other than ABCG2 inhibition could also be involved in the reduction of danofloxacin residues in milk by flaxseed. In fact, there are other biologically active components present in flaxseed at relatively high concentrations, such as alpha linolenic acid. However, to the best of our knowledge, no effect on drug transport of this fatty acid has been previously reported. In any case, it cannot be ruled out that a potential change in milk composition such as in lipid content due to diet supplementation with flaxseed [2, 14, 41] could affect the concentration of antimicrobials in milk.

Some other disturbing factors may also be present in our experimental design. For instance, our experimental diets include small variations in ingredients other than flaxseed in order to achieve nutritionally balanced diets (Table 1). However, as far as we are aware, indications of potential effects of these other ingredients on drug transport have never been reported in previous studies, although they cannot be completely ruled out.

Our findings are a new step toward the identification of food-drug interactions that alter drug secretion into milk. They could lead to improved adjustment of treatment regimens to guarantee efficacy and avoid the development of resistance. A change in drug secretion into milk, therefore, could have potential consequences for drug efficacy and amounts of milk residues. When danofloxacin is used for the treatment of mastitis, a reduced secretion of this antibacterial into the mammary alveolar space could influence its effectiveness, in particular for infections by extra-cellular pathogens. However, in the case of systemic diseases, such as respiratory infections, drug transfer into milk is undesirable. With regard to mastitis, danofloxacin may be effective when taking into account a C_max_/minimum inhibitory concentration (MIC) index of 3 or more against mastitis isolates with MIC < 0.12 μg/mL in the case of sheep [36]. The C_max_/MIC index of 15.3–18.3 in the flaxseed diet groups may ensure the effectiveness of our treatment regimen in mastitis.

Regarding residue formation, taking into account that the effect of isoflavones present in soy on danofloxacin residue in ovine milk was even higher (50% reduction) [18] than the effect of flaxseed in the present study (25–30% reduction), a combination of dietary enterolignan precursors with other phytoestrogens such as isoflavones could have a synergistic effect on drug residues in milk.

## Conclusion

In conclusion, flaxseed-enriched diets reduce concentration of danofloxacin in milk without changes in plasma concentrations. This manuscript highlights the relevance of flaxseed diets fed to livestock that modulate not only milk composition but also the concentration of antimicrobial residues.

## Abbreviations

AUC: Area under the concentration curve
BCRP/ABCG2: Breast Cancer Resistance Protein
C_max_: Maximum concentration
HPLC: High performance liquid chromatography
MDCKII: Madin-Darby canine kidney
MRT: Mean residence time
SDG: Secoisolariciresinol diglucoside
SEM: Standard error of the mean
T_1/2el_: Elimination half-life
T_max_: Time to peak concentration
